# A Feature Extraction Method Using Improved Multi-Scale Entropy for Rolling Bearing Fault Diagnosis

**DOI:** 10.3390/e20040212

**Published:** 2018-03-21

**Authors:** Bin Ju, Haijiao Zhang, Yongbin Liu, Fang Liu, Siliang Lu, Zhijia Dai

**Affiliations:** 1 College of Electrical Engineering and Automation, Anhui University, Hefei 230601, China; 2 National Engineering Laboratory of Energy-Saving Motor & Control Technology, Anhui University, Hefei 230601, China

**Keywords:** improved multi-scale entropy, multi-scale entropy, feature extraction, bearing fault diagnosis

## Abstract

A feature extraction method named improved multi-scale entropy (IMSE) is proposed for rolling bearing fault diagnosis. This method could overcome information leakage in calculating the similarity of machinery systems, which is based on Pythagorean Theorem and similarity criterion. Features extracted from bearings under different conditions using IMSE are identified by the support vector machine (SVM) classifier. Experimental results show that the proposed method can extract the status information of the bearing. Compared with the multi-scale entropy (MSE) and sample entropy (SE) methods, the identification accuracy of the features extracted by IMSE is improved as well.

## 1. Introduction

Rolling bearings are key components of rotating machinery [1]. Monitoring and diagnosing a bearing are significant measurements in ensuring rotation machines run steadily [2,3]. In recent decades, researchers studied many methods in condition monitoring and fault diagnosis for mechanical equipment [4,5,6]. These methods include traditional time- and frequency-domain analysis [7,8,9], discrete wavelet transform [10,11], fractal dimension (FD) [12,13], and empirical mode decomposition (EMD) [14]. An artificial intelligence (AI) method was also developed to perform mechanical fault diagnosis and running state monitoring, in order to improve the efficiency and effectiveness of fault diagnosis of machines [15]. However, in all, extraction of the status features for a health condition is one of the most critical steps [16].

Recently, researchers developed many kinds of feature extraction methods. Immovilli et al. used the Hilbert transformation and envelope analysis to identify the spectrum components and fault information of the fault bearing [17]. Another way is to use statistical analysis methods to obtain various characteristics of the signal, and these statistical indicators can be used for fault detection and classification [18]. The time-domain characteristics and frequency-domain characteristics of the vibration signal of bearing were applied to the neural network to establish an automatic motor bearing fault diagnosis system [19]. The results show that the neural network can be effectively used to diagnose the faults of various motor bearings and provide a new way for motor bearing fault identification. The nonlinear and non-stationary features of vibration signals add difficulty in obtaining feature information from the vibration signals of bearing. To solve this issue, Yang et al. proposed an intelligent fault diagnosis method based on the FD algorithm by using the SVM classifier [12]. This method, which is based on different dynamic mechanisms, uses fractional dimension algorithm to judge the working condition status of bearing and provides a valid diagnostic method for non-stationary vibrational signals. Sunil Tyagi et al. presented a DWT and SVM hybrid method for fault diagnosis of rolling bearings, which is more effective than the artificial neural network (ANN) classifier. Therefore, SVM has been widely used in bearing fault diagnosis [20]. Yan et al. proposed an approximate entropy for machine health monitoring [21]. This method shows that approximate entropy has the advantage of anti-noise ability and anti-wild point traits. Richman et al. proposed the conception of sample entropy (SE) [22]. This method overcomes low match degree and dependence of the time series length of the approximate entropy. Wang et al. combined SE with EMD for centrifugal pump fault diagnosis [23]. This combined method has higher recognition rate than single SE. Costa et al. proposed the concept of MSE for improving the drawbacks of SE [24,25]. This method is based on SE, applies the coarse granulation into SE, and well measures SE under different scales. The algorithm idea for SE is to find the maximum of the absolute value between the vector distances. However, this method is applied to measure the similarity between vectors, and ignores the second maximum points. To solve this problem, we propose an IMSE method. IMSE fully considers the global information for every distance vector. Experimental results show that IMSE can extract the fault feature information of bearings effectively.

The rest of this paper is arranged as follows. The theoretical background and algorithm steps of IMSE are introduced in Section 2. Section 3 gives two experimental cases for bearing fault identification. Section 4 gives the conclusions.

## 2. Methodology

### 2.1. Improved Sample Entropy

Based on the algorithm idea of approximate entropy, Richman et al. proposed SE [26]. However, SE has drawbacks in calculating the similarity of matrices because SE only considers local information. Therefore, an improved sample entropy (ISE) is proposed to extract the feature information of rolling bearings. The calculation steps of ISE are shown as follows:

(i) Time series are composed of *N* points, which can be written as $\left\{x_{i},\;i=1,\;2,\;\ldots ,\;N\right\}$. Pre-given parameters are embedded dimension *m* and similarity tolerance *r*. According to the original signal, a vector space with *m* dimensions is constructed as follows:
  (1) $$x(i)=\left[x_{i},x_{i+1},\cdots ,x_{i+m-1}\right]$$
  where *i* = 1, 2, 3, …, *N* − *m*.
(ii) The Euclidean distances between the *x*(*i*) and *x*(*j*) vectors can be written as *d*[*x*(*i*), *x*(*j*)], where *j* = 1, 2, 3, …, *N* − *m*; *j* ≠ *i*. Counting the number of *d*[*x*(*i*), *x*(*j*)] ≤ *r*, which could be expressed as *B_i_*, the ratio of *B_i_* to the total of distance number *N − m* − 1 is defined as ${B_{i}}^{m}$(*r*):
  (2) $$B_{i}^{m}(r)=\frac{1}{N-m-1}B_{i}$$
(iii) Calculating the mean value of Equation (2), we obtain the following:
  (3) $$B^{m}(r)=\frac{1}{N-m}\sum_{i=1}^{N-m}B_{i}^{m}(r)$$
(iv) When the dimension increases to *m* + 1, steps (i) to (iii) are repeated, and *B^m^*^+1^(*r*) can be obtained as follows:
  (4) $$B^{m+1}(r)=\frac{1}{N-\left(m+1\right)}\sum_{i=1}^{N-\left(m+1\right)}B_{i}^{m+1}(r)$$
(v) According to the definition of SE [26], ISE is given by the following:
  (5) $$ISE(m,r)=\underset{N\to \infty }{\lim}\left\{-\ln\left[\frac{B^{m+1}(r)}{B^{m}(r)}\right]\right\}$$
(vi) When *N* is a constant value, Equation (6) is given by the following:
  (6) $$ISE(m,r,N)=-\ln\left[\frac{B^{m+1}(r)}{B^{m}(r)}\right]$$

Obviously, the value of ISE is related to *m*, *r*, and *N*. ISE would vary with different embedded dimension *m* and similarity tolerance *r*. In this paper, *m* = 2 and *r* = 0.15*σ*, where *σ* is the standard deviation of raw signal [27,28].

### 2.2. Improved Multi-Scale Entropy

**Figure 1:**
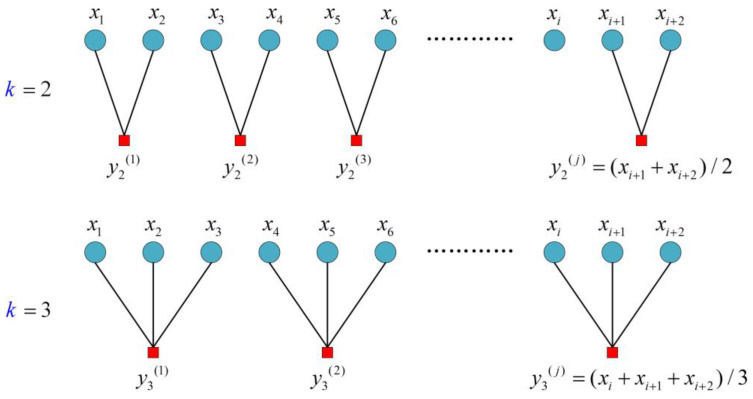
Coarse-grained process of time series.

For SE, the complexity of the time series is reflected only by a single scale. Costa et al. introduced the idea of coarse graining and proposed the MSE algorithm [25,29]. MSE is defined as the time series SE with different scales. If an entropy sequence decreases monotonically with increasing scale factor, the sequence complexity is relatively simple and vice versa. The calculation steps of improved multi-scale entropy (IMSE) are as follows:

(i) Scale factors *k* can be obtained from the process of coarse granulation. Setting up the coarse graining series with the original signal, we obtain the following:
  (7) $$P_{j}(k)=\frac{1}{k}\sum_{i=(j-1)k+1}^{jk}x_{i}，1\leq j\leq N/k$$
  where *k* is the scale factor with a positive value. When *k* = 1, the original series has not undergone coarse-grained analysis. Figure 1 gives the procedure of the time-series when *k* = 2 and 3, respectively. Figure 1 shows that the coarse grained process is based on the length of window function of the non-repetitive sliding averaging process, i.e., *k* [30].
(ii) For non-zero value *k*, {*x_i_*} is divided into a coarse granulation series, with length [*N/k*] and final coarse-grained series as {*P_j_*(*k*)}. We set the maximum scale at *k_max_* = 18. Based on the coarse-grain process for multi-scale analysis and calculating the ISE of [*P_j_*(1), *P_j_*(2),…, *P_j_*(*k_max_*)] with different scales [1, 2,…, *k_max_*], IMSE can be written as follows:
  (8) $$IMSE=\left[ISE\text{\_}1,ISE\text{\_}2,\ldots ,ISE\text{\_}k_{\max}\right]$$

In the coarse graining process of IMSE, by changing the scale factors, the pattern information of the original time series corresponding to scale factors can be obtained. Then, we calculate the ISE of the new time series. IMSE is defined as time series ISE with different scale factors.

**Figure 2:**
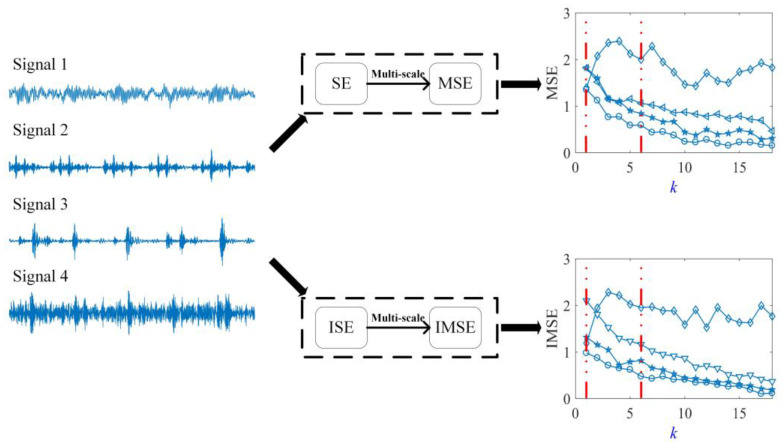
Divisibility performance of entropy curves between improved multi-scale entropy (IMSE) and multi-scale entropy (MSE).

The processing results of entropy curves using different methods are shown in Figure 2. Figure 2 shows that SE and ISE have not undergone multi-scale analysis, when *k* = 1. The divisibility of ISE is better than that of SE for rolling bearings on different vibration statuses. When *k* > 1, SE and ISE undergo multi-scale analysis to obtain MSE and IMSE. Compared with single-scale entropy, MSE and IMSE can separate different vibration statues of bearings more intuitively because of the introduction of scale factor *k*. When *k* ≤ 6, entropy curves for different signals are easily distinguished from one another.

### 2.3. Rolling Bearing Fault Diagnosis Based on IMSE Method

The vibration signal that is acquired from the signal acquisition device is always a one-dimensional time-series. When the defect of the rolling bearing occurs in different parts, the vibration signals are different, and thus, unable to reflect fully the status information of bearings. MSE is a feature extraction method that can measure the complexity of the time series [29]. In this paper, IMSE algorithm is proposed to address the problem of missing information in the calculation of the vector similarity of the traditional method, MSE.

**Figure 3:**
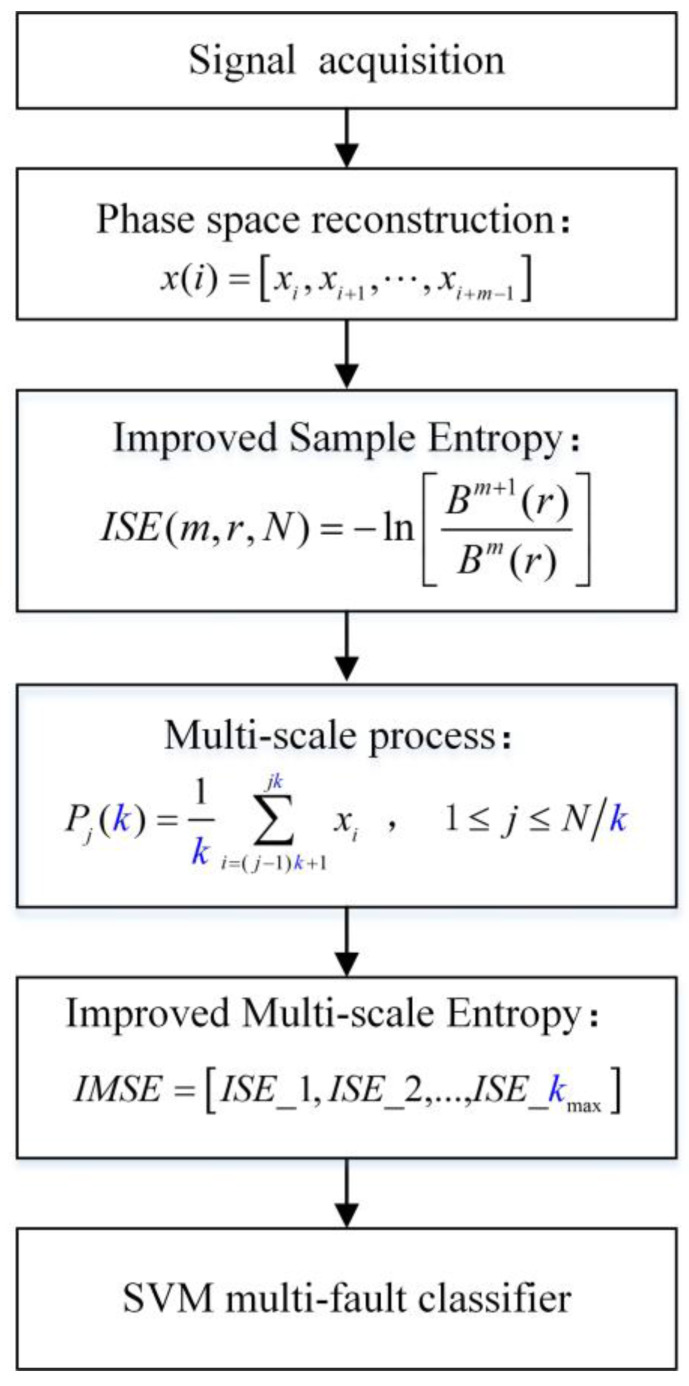
Flowchart of bearing fault pattern recognition based on IMSE and support vector machine (SVM) methods.

The acquired vibration signals are always overwhelmed by heavy background noise and accompanied by non-stationary and non-linear characteristics [31,32,33]. The key point of bearing fault diagnosis is the manner by which the fault feature information is extracted from the vibration signal with noises. The SVM classifier, which has good learning ability, has been used with IMSE algorithm to identify different vibration signals during different conditions [34,35,36]. The diagnosis flow chart of IMSE is shown in Figure 3. Overall, the bearing fault diagnosis based on IMSE algorithm can be summarized as follows:

(1) Signal acquisition;
(2) Phase space reconstruction;
  Reconstruct an *m*-dimensional vector space from the original signal *x*(*i*).
(3) Calculate the distance similarity between two different vectors;

First, we calculate the quadratic sum between the vectors of *x_m_*(*i*) and *x_m_*(*j*), then, we calculate the arithmetic square root for these two vectors.

(4) Calculate ISE parameters;

We count the distance *d*[*x*(*i*), *x*(*j*)] ≤ *r* on m dimension and denote as *B_i_*. Let *B_i_* divide the total of distance number *N* − *m* − 1 in their corresponding dimension, and denote the distance ratio as $B_{i}^{m}$. We calculate the mean value of $B_{i}^{m}$ and $B_{i}^{m+1}$, and denote their values as $B^{m}$ and $B^{m+1}$, respectively. We calculate the negative logarithm of the ratio of $B^{m}/B^{m+1}$.

(5) Calculate IMSE parameters;

Calculate the ISE of the coarse-grained vector that corresponds to different scales, then IMSE is obtained.

(6) Fault identification;

The obtained IMSE are taken as training dataset. Then, IMSE parameters from the testing dataset are fed into the SVM multi-fault classifier [35,37]. Finally, the fault patterns with the different fault patterns of rolling bearing are distinguished.

## 3. Application Cases Using IMSE Method

**Figure 4:**
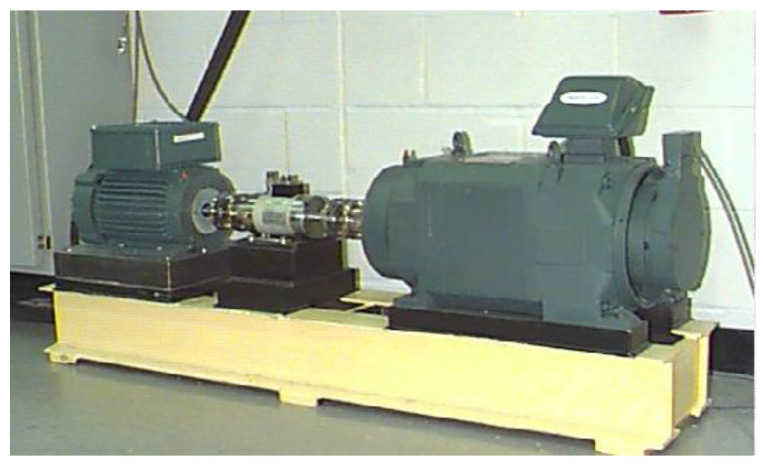
Bearing experimental table.

**Figure 5:**
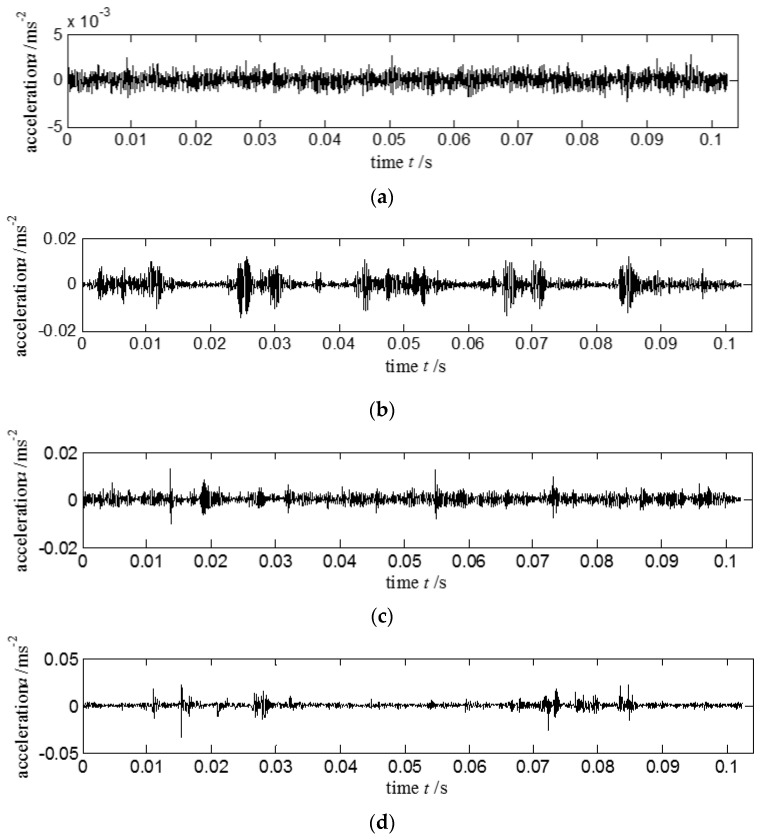
Different bearing status for the acquired vibration signals in time domain: (**a**) normal signal; (**b**) vibration signal with inner race-way fault; (**c**) vibration signal with outer race-way fault; (**d**) vibration signal with roller fault.

**Case I**: The acquired vibration signals with different fault types are downloaded freely from the bearing data center of Case Western Reserve University (CWRU) [38]. Bearing fatigue experiment equipment is shown in Figure 4. Table 1 shows the dimensions and parameters of the tested bearing. The tested bearing is a deep groove ball bearing, and the product type is 6205-2RS JEM SKF. The rotating speed of the tested bearing is *n_r_* = 1797 r/min, and the fault diameter is *D* = 0.533 mm. The status types of the acquired vibration signals are shown as normal, outer race-way, inner race-way and roller faults. The sampling points are *N* = 2048 and sampling frequency is *f*_s_ = 12 kHz. Acquired vibration signals in time domain with different status are shown in Figure 5. Figure 5a displays the vibration signal of bearing on normal condition. Figure 5b–d show the vibration signals of the bearing with inner race-way, outer race-way, and roller faults, respectively. The feature information of Figure 5b–d are difficult to observe. However, these figures indicate that the impact components obviously existed given several local defects occurring in the bearings.

MSE and IMSE of four bearing status are calculated. We selected 20 training and 30 testing samples, and different scale factors are trained as different eigenvalues. Therefore, the fault recognition rate under different eigenvalues is calculated. The calculation results are shown in Table 2. Table 2 shows that when the numbers of eigenvalues 4 and 7 are selected as the sensitive fault features, the recognition rates of MSE and IMSE have the same value. When the numbers of eigenvalues are changed to 5, 6, 10, 15, and 18, the fault recognition rate of IMSE is higher than that of the MSE. The reason is the possible existence of correlation and redundancy between the features of MSE and IMSE. Overall, Table 2 shows that IMSE has higher recognition rate than the MSE. When more eigenvalues are selected, more time is consumed. Therefore, the number of eigenvalues *k* is chosen as 5.

**Figure 6:**
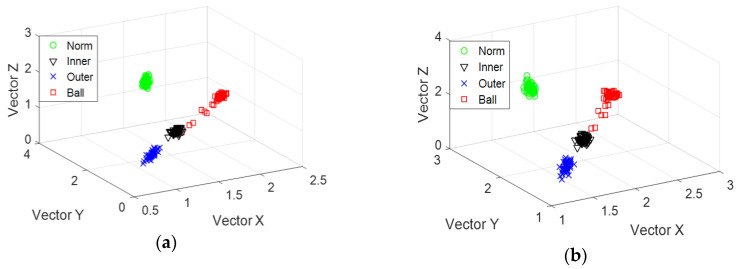
Classification results using two features: (**a**) MSE and (**b**) IMSE.

Different status information is calculated using MSE and IMSE methods. For each state, we considered 50 data sets and eigenvalue *k* = 5. Thus, the data dimension is 200 × 5. We took the first 200 × 3 data matrix as an eigenvector matrix. Classification results are shown in Figure 6a,b. Figure 6a shows an overlap between the roller and inner race-way faults. However, the overlapping phenomenon does not occur under different bearing statuses, as illustrated in Figure 6b. This result implies that different bearing statuses could be easily classified by using the IMSE feature.

**Figure 7:**
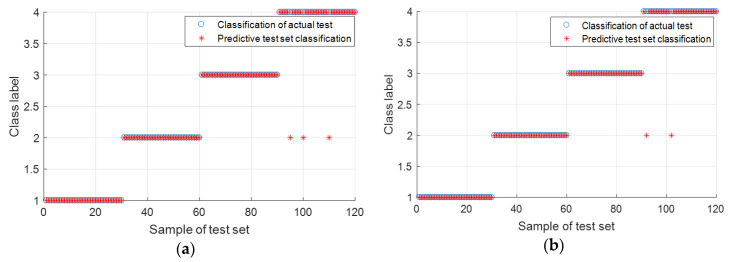
Fault recognition comparison chart: (**a**) MSE and (**b**) IMSE.

To be more intuitive in describing the superiority of IMSE, four state data are randomly selected, which included training and testing sets. Then, we calculated MSE and IMSE features. Eigenvector is inputted into the SVM system. Fault classification comparison results are shown in Figure 7a,b. These two figures show that the classification result processed by IMSE is slightly better than MSE.

Finally, different training samples are used as testing data to illustrate the influence of the number of training samples on identification results. The fault recognition rates of using different methods under different training samples are shown in Table 3. Table 3 indicates that when the training sample is selected as 20 and 30, the recognition rates processed by IMSE are slightly higher than those of SE and MSE. When the training samples are greater than 15, with the increase of training samples, identification rates of SE, MSE, and IMSE are gradually increased, and overall recognition rates of IMSE are slightly higher than those of the other methods.

In order to verify the anti-noise ability of MSE and IMSE, we applied these two methods on vibration signals with Gaussian white noise. The signal to noise ratio (SNR) of these added Gaussian white noises are 5, 10, 15 and 20 dB, respectively. These processed results using MSE and IMSE algorithms are shown in Table 4. From the definition of SNR, the larger the SNR, the smaller the noise mixed in the signal. It can be seen from Table 4 that the fault recognition rate of IMSE algorithm is slightly higher than that of MSE algorithm.

**Figure 8:**
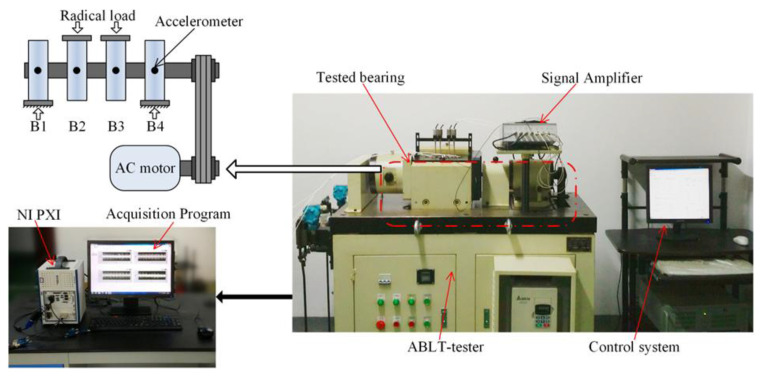
Experimental table of the tested bearing.

**Figure 9:**
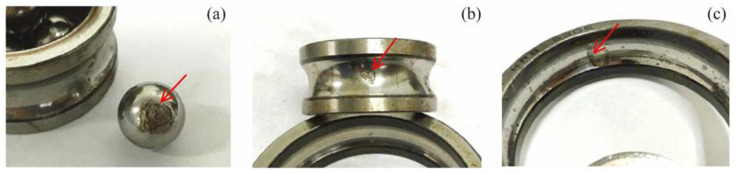
Tested bearing with different fault types: (**a**) roller fault, (**b**) inner race-way fault, and (**c**) outer race-way fault.

**Figure 10:**
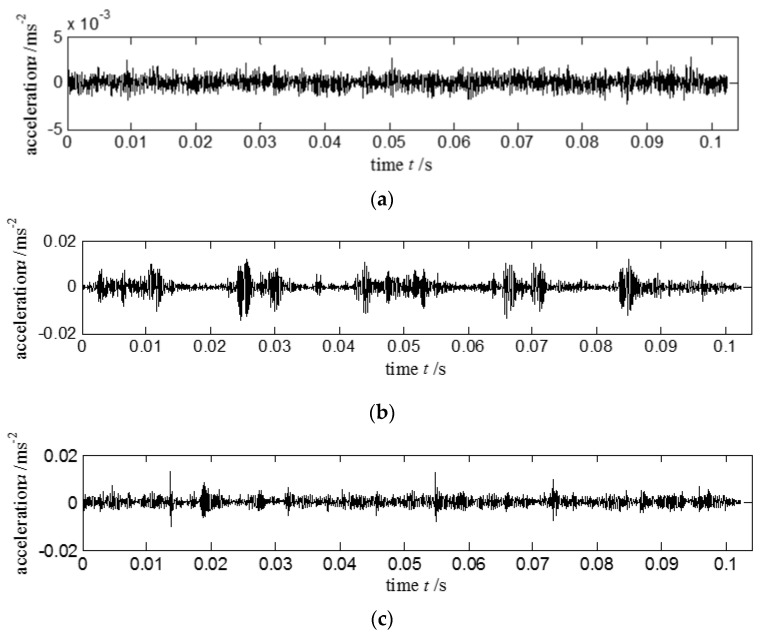

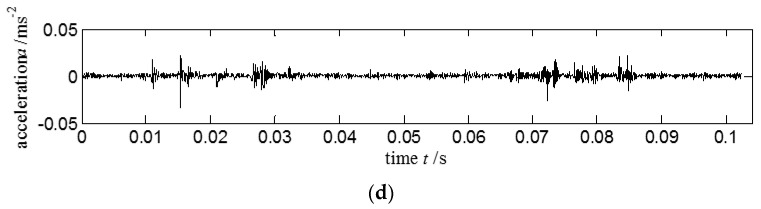
Different bearing statuses for the acquired vibration signals in time domain: (**a**) normal signal; (**b**) vibration signal with inner race-way fault; (**c**) vibration signal with outer race-way fault; and (**d**) vibration signal with roller fault.

**Case II**: This experiment combined LabVIEW with other data acquisition devices for acquiring vibration signals. Experimental test platform is shown in Figure 8. Detailed facilities included ABLT test platform, signal enhancement equipment, monitoring system, four tested bearings, and NI PXI acquisition system. Production type of the tested bearing is HRB6305. The tested bearing is embedded into the bearing sleeve. A three-phase electric motor provides the power. The bell is connected to the motor through the pulley. Radial load of the loading system *P* = 20 kN is loaded into the tested bearing. The rotating rated speed of the electric motor *n_r_* = 3000 r/min, the rated current *I_e_* = 6.3 A, and the sampling frequency *f*_s_ = 20 kHz. Tested bearing with different fault types is presented in Figure 9. Acquired vibration signals in time domain with different status are given in Figure 10. Figure 10a–d show the vibration signals of the bearing with normal, inner race-way fault, outer race-way fault and roller fault, respectively.

**Figure 11:**
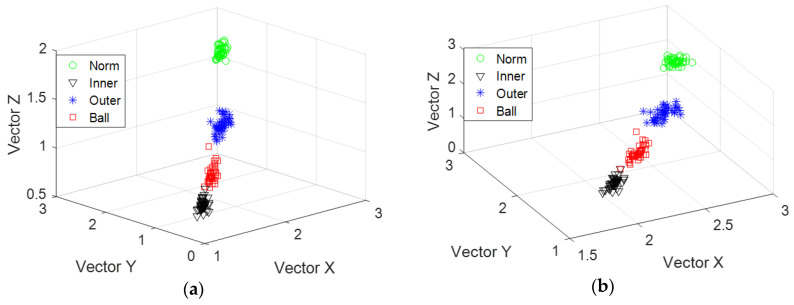
Classification results using two features: (**a**) MSE and (**b**) IMSE.

In this part, MSE and IMSE are applied to evaluate the recognition rate quantitatively. From Table 5, when *k* = 6, the recognition rate processed by IMSE is higher than the MSE. Fault recognition results using these two features are shown in Figure 11. Figure 11a shows the classification result using MSE. Figure 11b shows the classification result using IMSE. The two subfigures indicate that the obtained fault identifiability handled with IMSE is slightly better than that of the MSE. The experimental results showed that the fault recognition using IMSE is better than the MSE.

**Figure 12:**
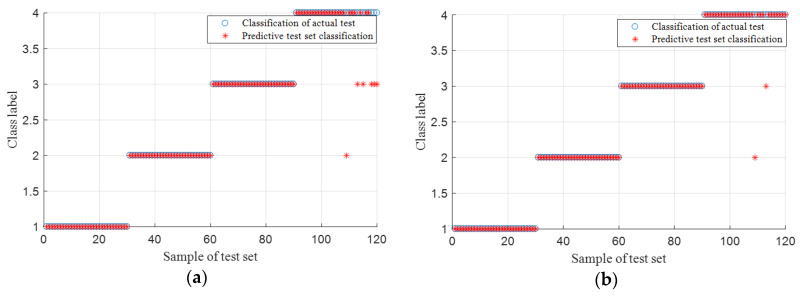
Fault recognition comparison chart: (**a**) MSE and (**b**) IMSE.

Classification results are illustrated in Figure 12a,b, respectively. Figure 12a shows that five test samples with roller fault are wrongly classified into the outer race-way fault, and one test sample is wrongly classified into the inner race-way fault. Figure 12b indicates that only two test samples with roller fault are wrongly classified into the outer and inner race-way faults. Experimental results of this case show the improvement in fault identification using IMSE compared with MSE.

Table 6 shows the quantitative analysis results. Here, different recognition rates using different classification methods are shown under different training samples numbers. Under different training samples numbers, identification rates processed by IMSE are higher than the other methods. The higher the fault recognition rate, the more accurate the detection of the bearing faults.

## 4. Conclusions and Discussion

In this paper, the IMSE algorithm was proposed for feature extraction and fault diagnosis of bearing. This algorithm is based on Pythagorean Theorem and the similarity criterion. The proposed method could decrease the information leakage problem of MSE when calculating the similarity of the vectors. Moreover, the algorithm is applied to extract the status features of rolling bearings. Experimental results imply that the proposed method has a higher fault recognition rate for rolling bearing fault diagnosis than that of MSE. The results of the two case studies show that the fault recognition rate depends on parameter *k* to some degree.

In the proposed IMSE algorithm, the uniform embedding was used. However, in article [39], non-uniform attractor embedding has been proved to effectively predict the changeable parameters by using fuzzy inference systems. In our future works, non-uniform attractor embedding might be a try in IMSE algorithm to improve the performance of the proposed IMSE method.

This paper mainly concentrates on investigating the IMSE algorithm. However, the selection of the proper scale factor spends lots of time, and it reduces the algorithm efficiency of the proposed IMSE method. To increase the adaptability of this algorithm, some intelligent algorithms might be introduced to improve the computational efficiency of the algorithm in order to allow adaptive selection of the parameters in the further works [40,41].

## Figures and Tables

**Table 1:** Physical dimensions of the tested rolling bearing.

Roller Diameter (mm; *d*_1_)	Pitch Diameter (mm; *d*_2_)	Number of the Rollers (*r*)
7.94	39	9

**Table 2:** Fault recognition rates using two classification methods under different eigenvalues.

*k*	4	5	6	7	10	15	18
MSE(%)	97.5	97.5	97.5	97.5	95.83	95	95.83
IMSE(%)	97.5	98.33	98.33	97.5	97.5	95.83	97.5

**Table 3:** Fault recognition rates using three features under different training samples.

Training Samples	5	10	15	20	25	30
SE(%)	96.67	95.88	95.43	95.83	97	97.5
MSE(%)	97.78	97.5	96.43	97.5	97	98.75
IMSE(%)	97.78	97.5	96.43	98.33	98	98.75

**Table 4:** Fault recognition rate of MSE and IMSE algorithms with different SNR.

SNR(dB)	5	10	15	20
MSE(%)	72.5	88.33	95	95
IMSE(%)	79.17	89.17	96.67	95

**Table 5:** Fault recognition rates using two features under different eigenvalues.

*k*	4	5	6	7	10	15	18
MSE(%)	95	95	95	93.33	93.33	93.33	93.33
IMSE(%)	95.38	97.5	98.33	97.5	97.5	96.61	97.5

**Table 6:** Fault recognition rates using three features under different training samples.

Training Samples	5	10	15	20	25	30
SE(%)	95	95	94.29	93.33	92	93.75
MSE(%)	95.56	95	95.71	94.17	94	96.25
IMSE(%)	95	95.63	98.57	98.33	98	97.5
